# Disparities in Adverse Childhood Experiences among Sexual Minority and Heterosexual Adults: Results from a Multi-State Probability-Based Sample

**DOI:** 10.1371/journal.pone.0054691

**Published:** 2013-01-23

**Authors:** Judith P Andersen, John Blosnich

**Affiliations:** 1 University of Toronto Mississauga, Department of Psychology, Mississauga, Ontario, Canada; 2 University of Rochester, Department of Psychiatry, Center for the Study and Prevention of Suicide, Rochester, New York, United States of America; 3 Department of Veterans Affairs, VISN-2 Center of Excellence for Suicide Prevention, Canandaigua, New York, United States of America; University of California, San Francisco, United States of America

## Abstract

**Background:**

Adverse childhood experiences (e.g., physical, sexual and emotional abuse, neglect, exposure to domestic violence, parental discord, familial mental illness, incarceration and substance abuse) constitute a major public health problem in the United States. The Adverse Childhood Experiences (ACE) scale is a standardized measure that captures multiple developmental risk factors beyond sexual, physical and emotional abuse. Lesbian, gay, and bisexual (i.e., sexual minority) individuals may experience disproportionately higher prevalence of adverse childhood experiences.

**Purpose:**

To examine, using the ACE scale, prevalence of childhood physical, emotional, and sexual abuse and childhood household dysfunction among sexual minority and heterosexual adults.

**Methods:**

Analyses were conducted using a probability-based sample of data pooled from three U.S. states’ Behavioral Risk Factor Surveillance System (BRFSS) surveys (Maine, Washington, Wisconsin) that administered the ACE scale and collected information on sexual identity (n = 22,071).

**Results:**

Compared with heterosexual respondents, gay/lesbian and bisexual individuals experienced increased odds of six of eight and seven of eight adverse childhood experiences, respectively. Sexual minority persons had higher rates of adverse childhood experiences (IRR = 1.66 gay/lesbian; 1.58 bisexual) compared to their heterosexual peers.

**Conclusions:**

Sexual minority individuals have increased exposure to multiple developmental risk factors beyond physical, sexual and emotional abuse. We recommend the use of the Adverse Childhood Experiences scale in future research examining health disparities among this minority population.

## Introduction

Adverse childhood experiences (e.g., physical, sexual, and emotional abuse, neglect, exposure to domestic violence, familial mental illness, substance abuse and incarceration) constitute a major public health problem in the United States [1]. A growing body of research provides evidence that adverse childhood experiences (ACE) can have systemic negative effects on health across the life-course [2]. The US Department of Health & Human Services reported that 3.6 million cases of childhood maltreatment received an intervention from Child Protective Services in 2010 [3]. The majority of these cases (86%) involved multiple forms of maltreatment. The most common types of maltreatment included neglect (78.3%), physical abuse (17.6%) and sexual abuse (9.2%). Parents were the most common perpetrators (81%). Nearly one-third of the instances of child maltreatment were from homes where a caregiver was an alcoholic and/or drug user (28.9%) or involved in domestic violence (25.7%). Moreover, studies suggest that certain minority populations, such as lesbian, gay and bisexual (i.e., sexual minority) populations, experience disproportionately higher prevalence of adverse childhood experiences.

### Defining Adverse Childhood Experiences

In a groundbreaking longitudinal study examining childhood risk factors associated with adult disease, researchers examined over 17,000 patients enrolled in medical treatment at the Kaiser Permanente Medical Care Program in San Diego [4]. In collaboration with the National Center for Chronic Disease Prevention and Health Promotion (CDC, Atlanta, GA), researchers defined a number of risky childhood exposures deemed Adverse Childhood Experiences (ACEs). These included growing up in a dysfunctional household environment (exposure to domestic violence, mental illness, alcohol or drug abuse, criminal behavior), abuse (physical, sexual, emotional), neglect (emotional and physical) and parental discord (caregiver separation or divorce). Results from this multi-wave study spanning more than a decade, revealed that two-thirds of the sample reported at least one ACE. Of those reporting one ACE, 87% reported at least one additional ACE category. Household dysfunction was highly prevalent, including parental discord (23.3%), substance abuse (26.9%) and at least one incarcerated family member (4.7%). Likewise, prevalence of emotional (10.6%), physical (28.3%) and sexual abuse (20.7%) was high [2], [4], [5].

The ACE project has significantly advanced prior research by including assessment of multiple childhood risk factors beyond sexual and physical abuse; it has drawn attention to the fact that adverse childhood experiences often occur in clusters rather than in isolation; and it has introduced the ACE scale for use in public health research. In 2009, members of the CDC, the World Health Organization, and public health officials from countries around the world met to outline a framework using the ACE scale as a standardized surveillance measure to assess the global burden of ACE on health [1]. Given the standardization and comprehensive nature of this measure, the ACE scale provides a common way to examine disparities in childhood maltreatment between heterosexual and sexual minority individuals in probability-based samples.

### Adverse Childhood Experiences and Health

Childhood maltreatment has been linked to a number of negative health outcomes including autoimmune conditions, cancer, heart disease, sexual and reproductive health problems, mental health (e.g., depression, panic, memory, impulse control and anxiety), risky health behaviors (e.g., smoking, drug and alcohol abuse, promiscuity), HIV, and somatic symptoms [2]. Felitti and colleagues reported a dose response relationship between the number of adverse childhood experiences and both health risk behaviors and chronic disease [4]. Specifically, odds of risk behaviors and mental health conditions increased exponentially with the number of adverse childhood experiences reported. For example, individuals reporting ≥2 or ≥4 adverse childhood experiences were significantly more likely to be smokers (OR = 1.5 & 2.2, respectively), depressed (OR = 2.4 & 4.6 respectively), or have attempted suicide (OR = 3.0 & 12.2, respectively). Similarly, for chronic disease outcomes, individuals reporting ≥4 or more adverse childhood experiences exhibited a twofold increase in odds of ischemic heart disease (OR = 2.2), any cancer (OR = 1.9), and stroke (OR = 2.4). Riley and colleagues documented a similar dose response relationship between severity of sexual and/or physical abuse and hypertension in data from the Nurses’ Health Study II [6]. Brown and colleagues found that individuals reporting ≥6 adverse childhood experiences died on average 20 years earlier than those without such experiences [7].

A number of studies have begun to explore the mechanisms by which adverse childhood experiences can affect health trajectories. In a 20-year prospective longitudinal study (N = 1,037) among a complete birth cohort of New Zealand residents, those who had experienced childhood maltreatment displayed significant risk factors for autoimmune and cardiovascular disease at age 32 years [8]. These individuals showed elevated levels of pro-inflammatory cytokines and C-reactive protein, a marker of inflammation and cardiovascular heart disease risk, even after adjusting for adult health practices and co-occurring life stress [8]. Changes in the structure and function of brain regions associated with learning, reasoning, memory and fear (e.g., hippocampus, prefrontal cortext, and amygdala) are associated with childhood adversity [9]. Neurobiological changes and disruptions in core stress response physiology, catecholamine secretion, corticotropin releasing factor, and cortisol have all been linked with childhood maltreatment [10] and are likely central mechanisms by which immune dis-regulation, cardiovascular disease, cancer and other chronic diseases are linked in a dose response manner to the number and severity of adverse childhood experiences.

The negative health impacts of adverse childhood experiences are clear. Anda and colleagues have named this body of evidence as the “face of a chronic public health disaster” [5]. Amidst this “disaster,” there are populations with disproportionately higher prevalence of adverse childhood experiences, such as sexual minority populations.

### Adverse Childhood Experiences Among Sexual Minority Individuals

A growing body of literature suggests a higher prevalence of childhood sexual assault (CSA), childhood physical assault (CPA), and emotional maltreatment among sexual minority individuals compared with their heterosexual peers. Rothman and colleagues conducted a comprehensive review of studies published between 1989–2009 that used probability (n = 25) and non-probability based samples (n = 50), which in total included nearly 140,000 sexual minority respondents. Collectively, prevalence of CSA for sexual minority women was 76% (the highest of all groups) and nearly 60% for sexual minority men [11]. These findings are alarming given that estimates of CSA in the general population are much lower (3% to 27% for women and 0% to 16% for men) [12]. More recent evidence has come from the 2004–2005 wave of the National Epidemiological Survey on Alcohol and Related Conditions (NESARC) [13]. Results from NESARC showed that sexual minority women were two times as likely to report child sexual abuse (CSA) than heterosexual women in their sample, and gay men were two times as likely to report CSA and childhood neglect compared to heterosexual males [13].

Research from the National Survey of Midlife Development in the United States (MIDUS) showed a higher prevalence of emotional maltreatment among sexual minority men (52.6%) and women (45.5%) compared to heterosexual men (36.5%) and women (37.2%), respectively [14]. Furthermore, Balsam and colleagues found higher prevalence of CSA, physical abuse, household violence, neglect, and psychological abuse among sexual minority individuals compared to their heterosexual siblings [15]. Similarly, Stoddard and colleagues found that 20.4% and 26.6% of lesbians reported physical and sexual abuse, respectively, compared to 10% and 15.7% of their heterosexual sisters [16].

Although the evidence of disparities in abuse between heterosexual and sexual minority individuals is mounting, there are limitations to prior research. First, research has yet to employ the ACE scale with a sample of sexual minority persons. Second, much of the current research on sexual minority persons has relied upon convenience samples [11]. Third, many studies combine gay/lesbian and bisexual populations to preserve statistical power, despite a burgeoning literature showing that many health risk indicators differ between gay/lesbian and bisexual groups [17], [18].

### The Current Study

To date, it is unclear if disparities exist between sexual minority and heterosexual individuals on dysfunctional household factors beyond emotional, sexual and physical abuse; factors such as those measured by the ACE scale. The goals of this study were to document among sexual minority persons the prevalence of early life stress as measured by the ACE scale, and to disentangle the prevalence of ACE among sexual minorities by distinctly analyzing gay/lesbian and bisexual groups. Using a large probability-based sample of sexual minority and heterosexual adults, this project tested the hypotheses that (a.) sexual minority adults would have increased odds of experiencing each of the eight ACE categories (i.e., familial substance abuse, mental illness, incarceration, parental discord, exposure to domestic violence, and physical, sexual and emotional abuse); and (b.) sexual minority persons would report a higher number of total ACE categories than their heterosexual peers.

## Methods

Data are pooled from three individual U.S. states’ Behavioral Risk Factor Surveillance System (BRFSS) surveys. Directed by the CDC, all U.S. states and territories annually administer surveys using standardized interviewing procedures with probability-based samples of non-institutionalized adults (http://www.cdc.gov/brfss/technical_infodata/surveydata/2010.htm). In 2010, Maine, Wisconsin, and Washington gathered self-reported sexual identity data from all respondents and used the CDC optional ACE module. Wisconsin administered the ACE module to its entire survey sample (n = 4,781). Maine and Washington each gathered two probability-based samples (i.e. “sample splits”), with each sample split receiving different optional modules. Maine administered the ACE module to one of two sample splits (3,791/8,132). Washington administered the ACE module to all respondents from split 1 and all respondents from two counties in split 2 (13,319/19,628). Thus, the analytic sample for the present analyses included all persons in the three states who were administered the ACE module (n = 22,071).

The CDC first introduced the ACE module for supplemental use in the BRFSS in 2009. The ACE module items were coded and categorized into eight domains of childhood adversities replicated from Bynum and colleagues [19]. In contrast to the CDC, a more conservative approach was used in the present analysis by recoding “don’t know/not sure” responses to be missing data rather than count them as a “no, the event did not happen” response. A count of ACE items was also generated ranging from zero to eleven, based on the affirmative answers to each ACE item.

Precise wording of sexual identity items varied slightly across the three states. Maine respondents were asked “How do you think of yourself?” whereas Washington and Wisconsin respondents were asked “Do you consider yourself to be…” Maine and Washington response options were “heterosexual or straight; homosexual, gay, or lesbian; bisexual; other; don’t know; refuse.” Wisconsin response options were “heterosexual, attracted to people of the opposite sex; gay [lesbian], attracted to people of the same sex; or bisexual, attracted to people of both sexes.” Sexual identity was recoded dichotomously into sexual minority (i.e., gay, lesbian, or bisexual) or heterosexual.

Demographic variables included age in years and sex (male/female). Educational attainment was dichotomized into those with a high school diploma or less versus those with at least some college education. Due to small numbers of racial/ethnic minorities among the gay/lesbian and bisexual groups, race/ethnicity was coded as white, non-Hispanic versus non-white and Hispanic persons.

Bivariate differences in demographic and ACE items were examined among the gay/lesbian, bisexual and heterosexual individuals using chi-square tests of independence. Due to multiple comparisons, a Holms Sequential Bonferroni adjustment was used among the blocks of ACE item pair-wise comparisons (e.g., gay/lesbian vs. bisexual; bisexual vs. heterosexual; gay/lesbian vs. heterosexual) [20], [21]. Logistic regression models adjusted for demographic characteristics and survey state (Washington as reference) were used to examine the association of sexual minority status with each ACE item. Given the dose response relationship documented in number of adverse childhood experiences and poor health [4], the rate of ACE was calculated by creating a variable for the total number ACE items experienced before the age of 18. A negative binomial regression model was used to estimate differences in the rates of ACE events among sexual minority persons when compared with their heterosexual peers [22]. Results are reported using incidence rate ratios (IRRs) and 95% confidence intervals, illustrating the ratio of rates of ACE events among gay/lesbian individuals compared with heterosexual individuals and bisexual individuals compared with heterosexual individuals. Due to the large sample size, 95% confidence intervals tend to be very narrow around point estimates because of increase statistical power. To reduce confusion in places where rounding would create the same value for point estimates and lower/upper bounds of the confidence intervals, all estimates from multivariable models are reported to three decimals. Missing data were handled with listwise deletion. All analyses were conducted using Stata/SE version 12. This study was approved by the ethics review board at the University of Rochester.

## Results

Within the sample, 1.2% self-identified as gay/lesbian, and 0.9% self-identified as bisexual. On average, sexual minority persons were significantly younger than their heterosexual peers (see Table 1). While gay/lesbian respondents were similar to heterosexuals in terms of proportions of men and women and racial/ethnic minorities, the bisexual group had higher proportions of females and racial/ethnic minorities. Whereas gays/lesbians had higher educational attainment than their heterosexual peers, bisexual persons had significantly lower levels of educational attainment than heterosexuals.

**Table 1:** Demographics, by Sexual Orientation.^1^

	Gay/Lesbian (GL)	Bisexual (B)	Heterosexual (H)
	(n = 262) n (%)	(n = 201) n (%)	(n = 20,250) n (%)
Age (Mean, SE)	52.9 (0.87)*	50.1 (1.39)*	56.6 (0.11)
Sex			
Female	144 (55.0)	144 (71.6)*	12,240 (60.4)
Male	118 (45.0)	57 (28.4)*	8,010 (39.6)
Education			
High school diploma or less	54 (20.7)*	93 (46.3)*	6,461 (31.9)
At least some college	207 (79.3)*	108 (53.7)*	13,761 (68.1)
Race/ethnicity			
White, non-Hispanic	30 (11.5)	35 (17.6)*	2,248 (11.2)
Non-white/Hispanic	231 (88.5)	164 (82.4)*	17,811 (88.8)

1 data from Wisconsin, Washington, and Maine.

* p<.05 when compared with heterosexual reference group.

Compared with heterosexuals, a significantly greater proportion of bisexual persons reported adverse childhood experiences across all categories (see Table 2), and gays/lesbians had significantly higher prevalence of all adverse childhood experiences except for parental separation/divorce. Gays/lesbian and bisexual respondents had largely similar prevalence of adverse childhood experiences except for parental discord.

**Table 2:** Adverse Childhood Experiences (ACE) by Sexual Orientation.

	Gay/Lesbian (GL)	Bisexual (B)	Heterosexual (H)	GLvs.B	Bvs.H	GLvs.H
	n (%)	n (%)	n (%)	*p* ^a^	*p* ^b^	*p* ^c^
ACE						
Household mental illness	68 (26.5)	69 (35.7)	3,395 (17.1)	.034	<.001*	<.001*
Household substance abuse	120 (46.5)	85 (43.8)	5,745 (28.8)	.569	<.001*	<.001*
Incarcerated household member	19 (7.3)	26 (13.4)	862 (4.3)	.032	<.001*	.018*
Parental separation or divorce	67 (25.8)	75 (39.3)	4,595 (23.1)	.002*	<.001*	.303
Exposure to domestic violence	62 (24.1)	43 (22.5)	3,040 (15.4)	.690	.007*	<.001*
Physical abuse	76 (29.3)	59 (30.3)	3,332 (16.7)	.833	<.001*	<.001*
Emotional abuse	123 (47.9)	92 (48.4)	5,846 (29.6)	.907	<.001*	<.001*
Sexual abuse	77 (29.7)	67 (34.9)	2,932 (14.8)	.245	<.001*	<.001*

a = p<.006 in Holm’s sequential Bonferroni adjusted significance level.

b = p<.03 in Holm’s sequential Bonferroni adjusted significance level.

c = p<.05 in Holm’s sequential Bonferroni adjusted significance level.

* = statistically significant according to adjusted significance level.

The majority of the group differences persisted in multivariate regression models after adjusting for sociodemographic characteristics. Compared with their heterosexual peers, gay/lesbian respondents had more than twice the odds of reporting physical, emotion, and sexual abuse (see Table 3). Bisexual persons had nearly three times the odds of reporting sexual abuse than heterosexual respondents. When looking at the total count of ACE items, gay/lesbian and bisexual respondents had nearly 1.7 and 1.6 times the rate of adverse childhood experiences compared with their heterosexual peers, respectively (see Table 4).

**Table 3:** Adjusted Odds Ratios of Adverse Childhood Experiences.

	Age	Sex	Education	Race/Ethnicity	Gay/Lesbian	Bisexual
	aAOR(95%CI)	AOR(95%CI)	AOR(95%CI)	AOR(95%CI)	AOR(95%CI)	AOR(95%CI)
Household Mental Illness n = 20,028	0.971*	0.576 *	0.793*	0.772*	1.854*	2.256*
	(0.969–0.974)	(0.531–0.624)	(0.729–0.862)	(0.682–0.874)	(1.499–2.294)	(1.644–3.094)
Household Substance Abuse n = 20,064	0.984*	0.785*	1.225*	1.059	2.086*	1.672*
	(0.982–0.986)	(0.737–0.837)	(1.147–1.309)	(0.960–1.167)	(1.623–2.680)	(1.247–2.242)
Incarcerated Household Member n = 20,128	0.958*	1.061	2.013*	1.789*	1.488	2.187*
	(0.954–0.962)	(0.923–1.220)	(1.752–2.313)	(1.509–2.121)	(0.891–2.486)	(1.398–3.420)
Exposure to domestic violence n = 19,895	0.986*	0.926	1.357*	1.644*	1.777*	1.330
	(0.984–0.989)	(0.855–1.003)	(1.251–1.472)	(1.473–1.835)	(1.326–2.381)	(0.939–1.884)
Parent Separation or Divorce n = 20,036	0.972*	0.883*	1.468*	1.317*	1.084	1.600*
	(0.970–0.974)	(0.824–0.946)	(1.366–1.576)	(1.190–1.457)	(0.811–1.447)	(1.169–2.181)
Physical Abuse n = 20,043	0.988*	0.978	1.124*	1.372*	2.019*	1.837*
	(0.985–0.990)	(0.907–1.056)	(1.038–1.217)	(1.229–1.531)	(1.535–2.655)	(1.340–2.519)
Emotional Abuse n = 19,858	0.981*	0.972	0.937	0.975	2.057*	1.922*
	(0.979–0.983)	(0.913–1.035)	(0.876–1.002)	(0.884–1.076)	(1.600–2.645)	(1.432–2.580)
Sexual Abuse n = 19,922	0.993*	0.338*	0.918	1.298*	2.592*	2.826*
	(0.991–0.996)	(0.308–0.371)	(0.842–1.001)	(1.150–1.464)	(1.962–3.425)	(2.072–3.856)

Note: All analyses are adjusted for survey state (Washington as reference group).

a AOR, adjusted odds ratio; CI, confidence interval; Reference group for sex, female; for education “at least some college”; for race, white; heterosexual is reference group for gay/lesbian and bisexual.

* = p<.05.

**Table 4:** Incidence Rate Ratios of the Number of Adverse Childhood Experiences.

	Number of Adverse Childhood Experiences
	aIRR (95%CI)
Age	0.983 (0.981–0.984)*
Sex	0.802 (0.772–0.834)*
Education	1.088 (1.044–1.132)*
Race/ethnicity	1.186 (1.118–1.259)*
Gay/lesbian	1.663 (1.428–1.936)*
Bisexual	1.584 (1.325–1.895)*

a IRR, incidence rate ratios; Reference group for sex, female; for education “at least some college”; for race, white; heterosexual is reference group for gay/lesbian and bisexual.

* = p<.05.

## Discussion

This study is among the few that have used a probability-based sample of sexual minority adults to examine early life stress, and it is, to our knowledge, the only one to date that has used the ACE scale. In addition to assessing multiple forms of abuse, the ACE scale provides assessment of household dysfunction (i.e., familial mental illness, substance abuse, incarceration, parental discord, and domestic violence). These variables have been largely unexplored in the literature comparing the early life experiences of heterosexual and sexual minorities. This is among the first reports to show that in addition to abuse, sexual minorities may report higher rates of household dysfunction such as familial mental illness, substance abuse, incarceration, and for bisexuals, parental discord.

In support of our hypotheses, our results suggest that sexual minority individuals had increased odds of exposure to each of the majority of adverse childhood experiences, and they reported a significantly higher rate in the *number* of adverse childhood experiences. This is particularly noteworthy in light of the evidence of the dose response relationship between adverse childhood experiences and poor health outcomes [4]. Sexual minority populations experience numerous mental health disparities, such as depression, anxiety and suicidal behavior [23]. Data about physical health disparities in sexual minority populations are limited, largely due to the lack of inclusion of sexual minority measures in population-based health surveillance surveys. However, disparities have been identified in asthma prevalence [24], [25]. It is unclear if adverse childhood experiences are major drivers of these disparities. In order to test causal explanations, it is crucial that future research focus on prospective studies that comprehensively measure variables such as gender nonconforming behavior, childhood adversity and the development of sexual orientation and sexual behavior over time.

The present findings on physical, sexual and emotional abuse corroborate literature outlining increased prevalence of these experiences among sexual minorities in comparison to heterosexuals [13], [14]. Specifically, sexual minority individuals in this sample had nearly twice the odds of experiencing physical, emotional and sexual abuse when compared to their heterosexual peers. Furthermore, by parsing gay/lesbian and bisexual groups, results indicated that bisexual individuals had almost three times the odds of experiencing sexual abuse than their heterosexual peers. This finding supports previous literature that suggests the examination of gay/lesbian and bisexual populations separately may be important for understanding particular experiences among each subgroup when comparing them to their heterosexual peers [17], [18]. For example, in this sample, lesbian/gay respondents did not differ from heterosexuals in reporting parental discord, whereas bisexual persons reported the highest prevalence. If lesbian/gay and bisexual persons had been combined into one group, that entire group would have seemingly reported higher prevalence of parental discord than the heterosexual group where the actual driver of the difference was only the bisexual group.

The etiology of these sexual orientation based disparities in childhood adversity is unclear. Some researchers posit that childhood adversity (particularly sexual abuse) may play a causal role in the development of same-sex preferences and or sexual minority identity [26], [27], [28]. For many reasons, studies that suggest abuse or dysfunction causes minority sexual orientation may be less apt explanations for the higher prevalence of such reports. First, there is an empirical disconnect between prevalence of abuse and prevalence of lesbian, gay and bisexual (LGB) sexual orientation among the general population. For instance, research from nationally representative data shows the prevalence of ACEs to be quite high, with estimates ranging from greater than 50% of respondents endorsing one ACE, more than 25% of respondents reporting at least 2 ACEs, 30.1% reported being physically abused, and 19.9% reported sexual abuse [4]. In terms of prevalence of LGB sexual orientation, the most recent nationally representative polling of the US population [29] showed that only 3.4% of the population identified at lesbian, gay, bisexual (or transgender). If abuse or familial mental illness, substance abuse, incarceration, or domestic violence (either alone or in combination) caused a child to become lesbian, gay or bisexual, there should be a much higher percentage of the population identifying as LGB. Second, the studies are based on cross-sectional data, which precludes causal inference. Third, not all sexual minority individuals in the samples were abused (i.e., if abuse causes LGB sexual orientation, then all LGB people should have reported abuse). Lastly, these studies did not examine a key variable, namely gender nonconforming behavior, which may explain differential abuse among sexual minority persons.

Gender nonconforming behavior is behavior in opposition to societal gender norms (e.g., a male who takes ballet lessons, a female who wears men’s clothing). LGB persons are, arguably, gender nonconforming in the very nature of their attraction to persons of same sex. While gender nonconforming behavior is not necessarily an indication of childhood sexuality, it is associated with sexual orientation in adulthood [30], [31]. Moreover, gender nonconforming behaviors are often recognized by adults before a child is aware of a sexual identity [32], [33], [34]. Evidence indicates that both adults and peer groups may resort to physical violence or abuse to censor gender nonconforming behavior or other indications of sexual minority status [35], [36]. In families experiencing dysfunction such as alcohol abuse and mental illness, a child with gender nonconforming behavior may more likely be targeted for abuse in this environment [35], [37]. Thus, rather than sexual abuse being causal of sexual orientation, unmeasured underlying factors, such as gender nonconforming behavior, may increase the likelihood of victimization of some children who later identify as a sexual minority [38], [39].

Another explanation for increased reports of familial dysfunction by sexual minority populations is a willingness among LGB people to disclose private, stigmatizing, or delicate information. Findings from several studies reported that a majority of LGB participants had attended psychotherapy, which may increase an individual’s recognition of family dysfunction and comfort in disclosing ‘taboo’ information [40], [41], [42]. Further, it is possible that, given the social stigma leveled against LGB identity, sexual minorities may spend considerable time reflecting on the meaning of identity, authenticity, and the ways in which developmental experiences may have shaped their lives [43]. So, for instance, it is possible that bisexual individuals who have experienced parental separation or divorce may be more likely to identify as a sexual minority given that the strictures and scripts of heterosexual norms for marriage already have been removed or edited in their schemas, and they may feel comfortable publically expressing their identity.

The need to discern the etiology of such targeted violence against sexual minority individuals is made all the more important given that early victimization is a risk factor for victimization in adulthood [44]. Combined with previous research, the current findings suggest that LGB youth may experience significant disadvantage early in their developmental trajectories given the higher prevalence of household dysfunction and familial victimization. This is to say nothing of the substantial evidence showing that sexual minority youth are more likely than their heterosexual peers to experience assault, abuse and bullying outside the home [37], [45]. The synergistic effects of familial and non-familial victimization among sexual minority populations are key areas for future research. Furthermore, there is a clear impetus for research about perpetrators of violence against sexual minority persons. Data about victimization come largely from victims’ self-reports, but the actual reasons for *why* a sexual minority person was selected for victimization lie more clearly with the perpetrator(s) of the acts.

Several limitations must be noted. First, despite being a large probability-based sample, the analyses are based on data from three states, which limits generalizability. Moreover, because the data were pooled across three states that administered the ACE scale to varying proportions of their total sample (e.g., sample splits or specific counties within sample splits), sample weights were not used. BRFSS sampling methodology may result in different forms of bias, such as the lack of inclusion of institutionalized persons and the inability to survey persons who have only cellular telephones and not a landline household phone. The self-identity measure of sexual orientation may introduce selection bias, in that the measure typically identifies respondents who are willing to disclose their sexual orientation. The average age of respondents in this study was in middle adulthood, so there may have been recall bias in reporting ACE items from before the age of 18. Furthermore, there may be unidentified cohort effects of how people disclose certain ACE. For example, for middle-aged adult respondents, parental discord may be more taboo given the era in which they grew up, versus young adult respondents who grew up in later decades when parental discord was more prevalent. Lastly, the sample size of sexual minority persons did not permit analyses to specifically examine gender and racial/ethnic minority groups.

In spite of these limitations, this study is currently one of the largest to examine multiple adverse childhood experiences in a probability-based sample of sexual minority adults and the only study to use the ACE scale. A benefit of this widely used ACE scale is that it contains multiple risk factors in addition to childhood sexual and physical abuse that may contribute to health outcomes. Including the ACE scale in future research among sexual minority individuals constitutes a valuable measure of developmental risk factors. While research is clearly needed to examine the longitudinal consequences of childhood adversity among sexual minority populations, there is an equal, if not more pressing imperative to *prevent* the maltreatment of sexual minority children and youth.
